# Disorganized attachment and defense: exploring John Bowlby’s unpublished reflections

**DOI:** 10.1080/14616734.2017.1380055

**Published:** 2017-09-27

**Authors:** Samantha Reisz, Robbie Duschinsky, Daniel J Siegel

**Affiliations:** ^a^ Department of Public Health and Primary Care, University of Cambridge, Cambridge, UK; ^b^ Mindsight Institute

**Keywords:** Disorganized attachment, self-regulation, segregated systems, defense, interpersonal neurobiology

## Abstract

Main and Solomon were the first to create a formal infant Strange Situation classification of attachment disorganization. Bowlby’s reflections on the underlying psychological processes of such behaviors, however, began early in his career, including the term “disorganization.” Most of these remained unpublished but are available through the John Bowlby Archive. Bowlby saw affective experiences as the source of the attachment behavioral system’s organization and regulation, and he introduced the term “effector equipment” to describe the emergent organization of attention, expectation, affect, and behavior to orchestrate responses to the environment. In his thinking, disorganization results from threat conflict, safe haven ambiguity, and/or activation without assuagement, which interfere with coordination and integration across a behavioral system. Bowlby’s unpublished writings also amplify his published work on segregated systems and defensive exclusion. Bowlby’s insights are relevant today and can provide greater background and clarity to current work, as researchers and clinicians consider the origins, manifestations, and meaning of disorganization.

## Introduction

Disorganized infant attachment is a topic that receives substantial attention from researchers and clinicians (e.g. Granqvist et al., 2017). Main and Solomon (1986, 1990), researchers based at the University of California, Berkeley, were the first to propose the formal disorganized attachment classification for the Strange Situation Procedure (Ainsworth, Blehar, Waters, & Wall, 1978). However, theorizing about the process of disorganization and attachment has a longer history that has value today, as empirical and clinical applications of attachment theory continue to expand. John Bowlby, the father of attachment theory, left an array of considerations of the behaviors later used by Main and Solomon to operationalize the disorganized classification. A small number of such reflections can be found in his published works (e.g. 1969, 1980). Most of his ideas, however, remain in his unpublished texts and correspondence housed at the Wellcome Trust Library Archive in London, United Kingdom. With the permission from the Bowlby family and encouragement from Main and Solomon, this article offers insight into those works.

Main and Solomon were naturally familiar with Bowlby’s published remarks on disorganization when they introduced the classification in 1990, and they have continued to point readers towards Bowlby’s published discussions (e.g. Solomon & George, 2011). However, Bowlby’s extensive notes were on the other side of the Atlantic and remained unpublished. This effectively meant that the wider context of Bowlby’s theorizing about disorganization has been missing from the literature, as Solomon, Duschinsky, Bakkum, and Schuengel (2017) have recently noted. As a result of this missing wider context, the remarks that Bowlby did publish – for instance, an important chapter on conflict and motor breakdown in Bowlby (1969, chapter 6) – have been difficult for readers to interpret effectively, consider clinically, or link to developments in the classification of infant attachment.

It is our hope to make these forgotten reflections accessible to researchers and clinicians through review of Bowlby’s unpublished written remarks. As such, this article adds to the excellent historical biographical literature on Bowlby’s work (e.g. Van Der Horst, 2011). This article also explores how Bowlby’s unrecognized insights might further current discussions about disorganized attachment today, such as different origins and pathways, connections to self-regulation, and implications for clinical work. We argue that these ideas from the Bowlby Archive are aligned with perspectives from the contemporary transdisciplinary field of Interpersonal Neurobiology (e.g. Siegel, 2017). While this framework formed after Bowlby’s passing, we believe he would have welcomed it as aligned with his own interdisciplinary way of thinking. Connecting past and present through links with Interpersonal Neurobiology, this paper demonstrates how Bowlby’s clinical acumen and theoretical rigor mean that his reflections can still contribute to discussions of disorganized attachment today.

We begin with a brief overview of disorganization and address the difficulties with terminology that have limited the recognition of Bowlby’s published reflections. Bowlby’s general theory of attachment disorganization will then be outlined, with an in-depth discussion of segregated systems and defensive exclusion. The article concludes by drawing out some implications relevant to future research and clinical practice.

## Brief overview of disorganized attachment

The Strange Situation Procedure, developed by Mary Ainsworth and colleagues (1978), is the gold standard assessment for attachment in infancy. In Ainsworth’s Strange Situation Procedure, a caregiver leaves the infant twice in a novel environment with interesting toys, first with a stranger and then alone, before returning. Infant behavior during the procedure is recorded, coded, and used to classify child–caregiver attachment. Ainsworth initially identified three patterns of attachment behavior. The secure pattern was characterized by the infant displaying distress on separation from the caregiver, pleasure on reunion, and a capacity to make use of the caregiver’s comfort to readily return to play. This was in line with Bowlby’s (1969) concept of the *attachment system* in which primate infants seek physical proximity and attention from their caregiver (their *attachment figure*) when they perceive threat or discomfort. Ainsworth also identified two insecure patterns of infant attachment. An insecure-avoidant pattern was characterized by infants masking their distress through focusing their attention on the external environment, such as on toys, and away from the caregiver. Ainsworth’s home observations indicated that these infants wished to gain the availability of the caregiver but seemed to know from experience that attempts to do so would be counterproductive, as they would likely be rebuffed if they displayed distress. The third pattern Ainsworth identified was resistant-ambivalence, in which infants show persistent distress and/or anger at the prospect of caregiver unavailability, such that they are often unable to return to play after reunion. The Ainsworth attachment classifications predict a wide variety of social, emotional, behavioral, and health outcomes even decades later (Ehrlich, Miller, Jones, & Cassidy, 2016; Sroufe, Egeland, Carlson, & Collins, 2005).

The Ainsworth classifications of attachment form coherent and comparatively discrete patterns that are predictable. Main and Solomon (1986, 1990) introduced an additional “disorganized” classification for the Strange Situation to encompass a variety of behaviors that appeared to reflect a disruption in the coherence of the infant’s strategy for seeking their caregiver when distressed. In formulating this new classification, Main and Solomon closely analyzed recordings of infants from both low-risk and high-risk samples, selecting certain behaviors that they clustered into seven indices based on their observable characteristics:

1. Sequential displays of contradictory behavior
2. Simultaneous display of contradictory behavior
3. Undirected, misdirected, or incomplete movements
4. Stereotypies, mistimed movements, and anomalous postures
5. Freezing or stilling
6. Display of apprehension of the caregiver
7. Overt signs of disorientation

In the Strange Situation, infants who display behaviors listed in the disorganized indices are rated for disorganization, and scores that reflect behaviors above a threshold level of intensity result in a disorganized classification (Main & Solomon, 1990).

Main and Solomon (1990) proposed that one pathway to disorganized attachment in the Strange Situation, though not necessarily the only one, would be if a child has a history of experiencing alarm with respect to their caregiver. This could be expected in a number of contexts, including abuse, family violence, or a parent whose unresolved trauma leads to disoriented or frightened behavior that frightens their child. The attachment system impels a child to seek their caregiver when alarmed, so experiences of the *caregiver themselves* as a source of alarm create conflict for the child between two incompatible motivation systems – approach towards and withdrawal from the caregiver. A child’s experience of this kind of motivational conflict was predicted by Main and Hesse to result in disruption of the attachment system in the Strange Situation and lead to the conflicted, disoriented, or apprehensive responses that Main and Solomon used to form the disorganized attachment classification.

Links between alarming caregiver behavior at home and disorganized attachment in the Strange Situation are well establishccounting for 13% of variance in disorganization (Madigan et al., 2006). Yet in recent years, there have been calls for renewed attention to the concept. Hesse and Main (2006) argued that it would be “a worthwhile endeavor for developmental psychopathology” to study different caregiving contexts and “compare these to the forms of D behavior exhibited by their infants” (p. 335). Solomon and George (2016) and Lyons-Ruth and Jacobvitz (2016) have likewise argued that attention to the different processes and behaviors implicated by disorganized attachment would be valuable for research and clinical work with infants (see also Beeney et al., 2016; Hollidge & Hollidge, 2016; Padrón, Carlson, & Sroufe, 2014; Solomon et al., 2017; Waters & Crowell, 1999). Bowlby’s conceptualization and theory of disorganization have clear value as the field moves forward in addressing such questions.

## Issues of terminology

A specific difficulty in recognizing and interpreting Bowlby’s reflections relevant to disorganization is that his terminology used to discuss conflict was diverse and unsteady, drawing from psychoanalytic theory, ethology, psychiatry, cybernetics, and neurology. More generally, terminology was a consistent issue for Bowlby across his professional life, hindering his ability to communicate and be understood by colleagues. The problem was compounded in public communication where Bowlby regularly simplified the ideas he presented, sometimes to the point of serious distortion, in order for the basic points to have a chance to be heard amidst hostile responses and misunderstanding (Riley, 1983; Thomson, 2013). In his unpublished notes, he writes evocatively and from clear personal experience, of the pain of rejection and ill-fit experienced by one holding “an idiosyncratic model of the world” (undated file cabinet notes from the 1950s, PP/BOW/H.10). Taken together, the complexity, speculative nature, and diffuse terminology of his thinking about disorganization meant that he offered only some of the fruits of these reflections in print. The engine room of his thinking about conflict, incompatibility, and breakdown remained largely hidden from view, and away from criticism and misunderstanding. This includes a good number of unpublished works of theoretical speculations, as well as complete and incomplete articles, and files upon files of relevant notes and observations. With encouragement from the Bowlby family, the second author is presently editing a selection of the completed but unpublished works for publication.

### Origins of the term “disorganization”

Close examination of texts from the early 1970s suggests that Main inherited the term “disorganization” indirectly from Bowlby via her graduate study with Ainsworth (see Appendix for a timeline; Duschinsky, 2015). Bowlby explicitly introduced the concept and emphasized its value in his seminal article *Separation Anxiety* (1960). There, Bowlby states that he took the concept of disorganization from the neurologist Kurt Goldstein, who had been making use of a commonly used concept among neurologists of the 1940s and 1950s. “Disorganization” was a term that had been used quite widely by neurological researchers interested in strong affect as a potentially overwhelming physiological experience (for a review, see Leeper, 1948). Goldstein argued that certain affects, such as anxiety, anger, awe, and ecstasy, could be so intense and absorbing that the organism could become disoriented, lost in the affect, and unable to respond behaviorally to the demands of the situation (Goldstein, 1951). Building on Goldstein, Bowlby (1960) added that grief also results in such a state of behavioral disorganization.

Bowlby’s observations of behavioral disorganization began early in his career. His unpublished notes from as early as 1939 contain descriptions of disoriented, overwhelmed, and fragmentary forms of interpersonal behavior that he observed among the evacuated children and the combat veterans he had worked with clinically during World War II (unpublished manuscripts on the psychology of evacuation, c. 1939–1942, PP/BOW/C.5/4/1; Bowlby & Soddy, War Neurosis Memorandum, British Army, 1940, PP/BOW/C.5/1). Discussions of the evacuated children were included in the second book of his seminal trilogy, *Separation* (1973), many years after his observations and attachment theory had already been outlined. In the 1950s, Bowlby’s colleague James Robertson had movingly documented disoriented, overwhelmed, and fragmentary behavior in children who had been institutionalized in hospital and their behavior on returning home (e.g. Robertson, 1953, 1958; see also Bowlby, 1973, and version 1 of a large unpublished book manuscript reflecting on Robertson’s observations, c. 1956, PP/BOW/D.3/1). Those same behaviors were also recognizable in some noninstitutionalized children following brief separation from their caregivers (Robertson, 1953, 1958). Bowlby published a paper in 1960 intended for a psychoanalytic audience based on his observations of these behaviors in his clinical practice with families, which were similar to those of other clinicians working with child patients with histories of trauma (e.g. Fraiberg, 1982). Having emphasized the value of the concept of “disorganization”, he then promised, “this is a concept to which we shall be returning in a paper to follow” (Bowlby, 1960, p. 110). The promise was left unfulfilled, eliciting letters from readers requesting more detail about this idea of “disorganization” and why Bowlby thought it so important (e.g. correspondence with the Dutch Psychoanalytic Society, 1963, PP/BOW/B.5/20).

Bowlby did continue to apply the concept of disorganization in his published work. In *Attachment* (1969), he stated that one of his “main interests” was the study of “the conflicts arising when two or more incompatible systems are activated at once” (p. 174). He also restated the argument that behavior can become uncoordinated in the context of certain intense emotions: “Above a certain level, however, efficiency may be diminished; and, when in an experimental situation total stimulation is very greatly increased, behaviour becomes completely disorganised” (pp. 96–7). Such overwhelming intensity is specifically expected in the context of conflicts between strong motivational systems, and “in some cases, indeed, the behaviour that results when two incompatible behavioural systems are active simultaneously is of a kind that suggests pathology” (pp. 96–7). This conceptualization has clear connections to the disorganized behaviors and classification later outlined by Main and Solomon (1986, 1990).

The behaviors in the Main and Solomon (1990) indices are not all “disorganized” per se in the Goldstein/Bowlby sense of the term, which described disruption of coherence at a motor level. This is a source of terminological complexity and, in fact, Main and Solomon (1990) alerted readers that their chosen term had connotations that were not fully aligned with the phenomena they intended to capture – they explicitly state that “our category title is still not satisfactory” since the “apprehensive movements” that comprise Index VI (displays of apprehension towards the caregiver) do not display disruption or contradiction at a behavioral level (p. 133). Indeed, awareness of the caregiver as a threat can elicit behavior that is environmentally responsive and smoothly sequenced. Main and Solomon (1990) go on to state, “signs of apprehension may seem less disorganized or disoriented than many of the other behaviour patterns” (p. 136). Despite this, they conclude that “‘disorganized/disoriented’ still seemed an acceptable descriptive heading” (p. 136) to describe phenomena related to an inferred disruption at the level of the child’s attachment response (Duschinsky & Solomon, 2017). Nonetheless, Goldstein, Bowlby, and Main and Solomon have substantial overlap in their investments in the concept, using it to mean an affective and motivational predicament that disrupts behavioral sequencing and environmental responsiveness. However, one lesson from examining the origins of the concept of disorganization is the importance of considered and careful use of terminology about behavior, psychological process, and classification that matches intended meaning, rather than assuming that the term “disorganized” is self-evident in its meaning (Duschinsky & Solomon, 2017). Soon after the end of the Second World War, Leeper (1948) was already warning the neurological research community that the term was ambiguous and ripe for contributing to misunderstandings if adequate definition was not provided.

### “Disorganization” and the Strange Situation

Observations of disorganized behavior in the context of attachment-related distress were the next major step towards the creation of a disorganized classification. In a book chapter written in the years after completing her doctorate under Ainsworth, Main (1977) reported that she had begun collecting instances of “odd” or “disorganized” behavior in the Strange Situation. This collection would grow and develop over the next decade into the Main and Solomon indices.

Bowlby was very interested in Main and Solomon’s work when they began their study of conflicted, disoriented, and apprehensive child behaviors in the Strange Situation. In a 1978 lecture to the Canadian Psychoanalytic Society, Bowlby reported on the experience of watching tapes of behavior in the Strange Situation with Main. He described his fascination that on reunion “instead of approaching his mother, [a child] placed himself facing into the corner of the room, as though complying with a punishment, and then knelt down with his face to the floor” (1978/1988, p. 61). Bowlby’s personal notes from discussions with Main in March of 1978 (PP/BOW/H.78) report his curiosity that these conflict behaviors displayed by some infants in the Strange Situation were also being observed in the behavior of abused toddlers towards their caretakers in nursery by Main’s graduate student, Carol George (George & Main, 1979). As Main’s research continued, Bowlby described her work as “striking” and expressed public acceptance of the disorganized/disoriented attachment classification as an addition to Ainsworth’s procedure (Bowlby, 1988, p. 147).

Bowlby believed that the behaviors identified by Main and Solomon were likely “of great clinical concern” (1988, p. 124). In contrast to Main and despite his promise from the 1960s, Bowlby did not train his focus on the concept of disorganization nor did he attempt to operationalize it. However, he did not regard disorganization as an undifferentiated state. Indeed, he described disorientation, freezing, stereotypies, and approach–avoidance conflict as “deviant patterns” (1988, p. 141). In using the concept of patterns, Bowlby was mindful of a key difference from Ainsworth’s relatively discrete patterns of attachment. In the margins of his personal copy of Main and Solomon’s (1986) chapter, *Discovery of an insecure-disorganized/disoriented attachment pattern*, he wrote that the authors would have done better to call it a “status” because the unitary term “pattern” may result in confusion if readers interpret it in the Ainsworth sense (PP/BOW/J.7/6). In this marginalia, he observes that Main would likely agree with this reasoning, since she had indicated to him in a discussion on the 12 March 1986 that, in her view, “‘Trauma to the attachment system causes disorganisation of behavior but does not create a new category’” (PP/BOW/J.7/6). This, again, highlights difficulties around terminology. Bowlby’s main issue with the language of “new category” was that categories suggest discreteness and a unitary process, which was not necessarily the case with disorganization.

In contrast to the Ainsworth categories, children who showed one kind of behavior suggestive of motivational conflict could very well display others as well. It was thus difficult to discern the cause of any specific behavioral expression of disorganization because “children who show one of these more pathological responses tend to show others” as well (version 2 of an unpublished book on the Robertson observations entitled *Protest, Despair & Detachment*, 1965, PP/BOW/D.3/38). The presence of different kinds of disorganized behaviors did not necessarily imply to Bowlby that the behaviors shared the same root cause or occurred as a result of the same process (Solomon et al., 2017). Bowlby and Robertson suspected that different adverse circumstances and experiences interacted with each other, making additional behaviors more likely, thus producing a diverse range of determinants and behavior (c. 1965, PP/BOW/D.3/38).

Ainsworth shared Bowlby’s view. In a letter to John Gerwitz in August 1968, which was copied to Bowlby, Ainsworth wrote:

I do agree that there are varied indices of attachment, and my data suggest that these are not necessarily highly correlated. I also tend to agree that the approach behaviours are more stable indices of attachment than are the “disorganization” responses – perhaps because there may be more diverse determiners of disorganization behaviour than there are for approach behaviour to specific persons. I think it will require much more research to ascertain how “disorganization” responses relate to the more “positive” components of attachment. (PP/BOW/K.4/12)

These ideas about the causes of “disorganized” infant responses to the caregiver were stated again in Ainsworth’s (1972) published reply to Gerwitz’s criticisms of the validity of the Strange Situation, written whilst Mary Main was her doctoral student. Main and Solomon would also later observe that there diverse determiners of the different behaviors they were using to index disorganized attachment, in agreement with the earlier observations of Bowlby, Robertson, and Ainsworth. All suspected that in some way, these behaviors, though not necessarily interchangeable in their meaning, were concerning in representing some kind of disruption of emotional self-regulation, likely in the context of some problem facing the child–caregiver relationship.

## Bowlby’s theory: self-regulation and disorganization

Attachment and self-regulation are intricately interconnected (e.g. Schore, 2001; Schore & Schore, 2008; Siegel, 2017). Seeking proximity to their caregiver is a common and coherent strategy in infants for regulating distress. Bowlby’s unpublished writings include a rich and distinctive theorization about incompatible motivational responses and their consequences for behavior and emotional regulation. These ideas are pertinent to current discussions about the meaning of the disorganized attachment classification and the specific psychological processes involved (e.g. Lyons-Ruth & Jacobvitz, 2016; Solomon et al., 2017).

### Effector equipment

The attachment behavioral system in humans infants consists of a repertoire of precursor behaviors that mature into the components of a coordinated and regulated system (Bowlby, 1960, 1969). The key elements described by Bowlby (1960) were attending to the caregiver in the present (*attention*), expectations from past experience with the caregiver (*expectation*), crying when distressed and smiling for affection (*affect*), as well as protesting when potentially separated and seeking proximity (*behavior*). Experiences with the caregiver over the course of infancy usually allow these four components to consolidate into an integrated attachment behavioral response, particularly between 9 and 18 months (Bowlby, 1960; Bowlby, in Tanner & Inhalter, 1960). Ainsworth (1967) explained that a baby

does not somehow become attached and *then* show it by smiling at the loved person and crying when she leaves him. He gradually becomes attached through smiling and crying – and through adjusting his posture to his mother, suckling her breast, looking at her, listening to her, vocalising when she talks to him, scrambling over her. (p. 350)

Building on Ainsworth’s characterization, in his book *Attachment* (1969, p. 180), Bowlby described the process of becoming attached as the gradual incorporation of component responses into a goal-corrected system that is organized through experiences with the target of that system. He offered *effector equipment* as a concept to refer to the elements of the meta-behavioral system that orchestrates attention, expectation, affect, and behavior within a specific behavioral system (e.g. attachment) and determines the extent to which the system is flexibly responsive to the environment (1969, p. 49). Effector equipment thus regulates and integrates the attachment behavioral system.

Bowlby’s (1969) concept of effector equipment can be considered as a specification of one of the tasks Freud (1915/2001) assigned to the ego, which today might be identified as an aspect of executive function central to self-regulation and integration (Siegel, 2012, 2017). Bowlby introduced the term “organization” in Bowlby (1969) in reference to either this (1) process of assembly of the attachment system or (2) its behavioral product. This provided a technical definition of the term, though with the very unfortunate ambiguity between *process* and *product* that attends any word in English ending in “-ization.” This is another example of terminology obscuring meaning, as this wording would later lead to ambiguity regarding whether disorganization meant either or both (1) the result of not being able to assemble and consolidate an organized goal-corrected system and (2) having an organized goal-corrected system that is *then* put into a state of disorganization. This question has continued to be an issue in attachment research and links into the larger psychological question of state versus trait, which has quietly plagued discussions of disorganized attachment (Zeanah & Lieberman, 2016).

In Bowlby’s conception, developmental anomalies can be expected in the coordination of attention, expectation, affect, and behavior because integration is undermined when there is no one available around whom the attachment system can be organized. Bowlby (1953) predicted that the perceived unavailability of the caregiver in the context of alarm had a special capacity to lower the threshold of susceptibility to disorganization (p. 271). This prediction would be made again and evidence surveyed half a century later by Sroufe (1996) in a chapter on emotional development. Bernier and Meins (2008) further expanded this approach to offer a synthesized threshold model that aimed to explain why certain children seemed more vulnerable than others to disruption of the attachment system and display of conflicted, disoriented or apprehensive behaviors in the Strange Situation. Their model asserts that the threshold for disorganization varies between children as a function of genetic and social–environmental risk factors. The concept of effector equipment is well aligned with this conceptualization because of the similarity in how both explain the internal regulation of attachment and its responsivity to the environment.

### Pathways to disorganization

Bowlby’s unpublished reflections can add to the proposals of Main and Solomon (1990), Sroufe (1996), and Bernier and Meins (2008) regarding pathways to disorganization. In a 1957 manuscript and in later undated notes focused on conflict, Bowlby (PP/BOW/H.10) theorized that a behavioral system that was already organized would be prone to be undermined especially in three circumstances, though there is no indication that Bowlby saw these as mutually exclusive or as exhaustive. The first is where an expected source of safety is also clearly associated with threat. We term this as *threat conflict*. A second situation is where signals about safety are ambiguous, even without cues for threat. We term this *safe haven ambiguity*. One of the few published mentions of these two pathways occurred in *Separation* (1973), where Bowlby discussed the relative – though not absolute – distinction between them. He emphasized that “it is no less natural to feel afraid when lines of communication with base are in jeopardy than when something occurs in front of us that alarms us” (p. 119). The third situation in which Bowlby expected disruption to the attachment system to occur was when a strong motivation was intensely activated for a long time without assuagement, such as the child’s desire for their caregiver in the context of institutionalization. In print, he wrote: “As the sum of such disappointment mounts and hopes of reunion fade, behavior usually ceases to be focused on the lost object. Instead, despair sets in and behavior, lacking an object towards which to be organised, becomes disorganised” (1961, p. 334). We term this *activation without assuagement*. These three potential pathways described by Bowlby suggest how an activated attachment system that is met with contradiction, ambiguity, or a lack of assuagement can be undermined and, ultimately, become disorganized.

With due conceptual and terminological caution, Bowlby’s three pathways to disorganization can be placed in dialogue with later developments in the field. Bowlby’s (c. 1950s, PP/BOW/H.10) first pathway, threat conflict, suggests that approach–withdrawal conflict in relation to a caregiver can disrupt the functioning of the attachment system in infancy, though sophisticated strategies could be developed to handle such conflict later in development. This point of Bowlby’s agrees with Main and Solomon (1990) who argued that repeated experiences of conflict between attachment and fear in relation to the caregiver would be one pathway to disorganization in the Strange Situation. The observation or inference of motivational conflict between approach and withdrawal is also core to many of the indices used to classify infants as disorganized in the Strange Situation (Main & Solomon, 1990).

The second potential pathway to disorganization discussed by Bowlby (c. 1950s, PP/BOW/H.10) was *safe haven ambiguity*. Highly ambiguous signals about safe haven availability have the potential to be disorganizing and such ambiguity could occur even where the caregiver is not threatening, is present, and there has been no major separation. This point is also mentioned in passing by Main and Solomon (1990) and was later elaborated by Lyons-Ruth (2007). Lyons-Ruth has operationalized and found empirical support for a pathway to disorganized attachment in the Strange Situation among infants whose caregivers engage in disrupted safe haven communication. This pathway is of particular interest because it can be expected to occur in the absence of threat conflict.

Activation without assuagement was the third possible pathway to disorganization proposed by Bowlby (c. 1950s, PP/BOW/H.10). In this situation, disorganization becomes probable when the attachment system is active without assuagement for a long time. Solomon and George (2011) have highlighted this point as particularly significant because it suggests that care or custody proceedings involving sustained separation from a parent can themselves result in the disorganized behaviors in the Main and Solomon indices (1990). This renders the use of disorganized attachment as an assessment in care or custody proceedings potentially invalid as a measure of the history of the child–caregiver relationship, as disorganization may be the unintended result of the proceedings themselves. However, this is not a point that has received direct empirical scrutiny, and Bowlby’s reflections further highlight the need for more applied research in this area, despite the challenges of such research.

### Disorganization as a breakdown of regulatory processes and defenses

Bowlby suggests that an organism that experiences fear that disrupts the attachment system, such as in the situations described above, can be anticipated to suffer from “traumatic difficulty in cortical incompatibility of sense data” (PP/BOW/H.10, notes from a file tagged ‘Theory of Defence 1960–1963’). The trauma results in the components of the attachment system – attention, expectation, affect, and behavior – coming apart from one another. For instance, attention may come apart from the others as disorientation; the intensity of distress may overwhelm the ability of these components to coordinate; and behavior may demonstrate a contradiction between distressed desire for comfort from the caregiver and the expectation of rejection. For Bowlby, integration blockages would likely have relational, experiential, and neurological aspects, though these need not always be symmetrical or correspond neatly. This agrees with later evidence surveyed by Siegel (2012) that the compassionate caregiver–child communication and connection that lead to secure attachment seem to be the experiential basis for nurturing the child’s developing neural integration.

More generally, Bowlby’s conceptualization fits strikingly well with the field of interpersonal neurobiology, which views the mind in the context of an emergent, self-organizing, “embodied and relational process that regulates the flow of energy and information” (Siegel, 2012, p. 7; also see, 2017). Optimal self-organization results from links between differentiated elements of a system that are coordinated and balanced through “*integration,*” the same term Bowlby used for this process (Bowlby, c. 1986). Reflecting Bowlby’s emphasis on the importance of early traumatic experience, childhood trauma has been situated by studies in Interpersonal Neurobiology as a relational impediment to experiential and neurological integration (Schore & Schore, 2008; Siegel, 2012; Teicher, 2007), which is then reflected in a child’s attentional processes, expectations, affects, and behavior.

In his unpublished writings from the 1950s, Bowlby (PP/BOW/H.10) uses the breakdown of avoidance to illustrate the disorganization of defense mechanisms. In avoidance, attention is directed away from internal and external attachment-related cues, which reduces displayed affect and raises the threshold for activation of attachment behavior (Bowlby, 1960; Main, 1979). However, avoidance can become challenging if the individual experiences incompatible and strong motivational tendencies, confusing or ambiguous input about threat, or strong activation without assuagement. Someone whose effector equipment remains functional has

a flexible use of his behavioural repertoire, and an ability to process competing and conflicting information. By contrast, a brittle person shows little flexibility and responds to changing and stressful situations either by persevering rigidly in his original response or else by becoming disorganised. (Bowlby, 1969, p. 363)

This same concept is discussed in Interpersonal Neurobiology and elaborates to describe how linkage and communication between differentiated mental systems keep attention, expectation, affect, and behavior from either becoming too rigid or too chaotic (Siegel, 2012, 2017). Bowlby (1969) presumed that the form of conflict, disorientation, or apprehension shown by a child could be expected to differ predictably as a function of which defense mechanism was overwhelmed or weakened. Thus, the breakdown of avoidance would not look the same as the breakdown of a dissociative response or of preoccupied fixation on the caregiver, which Bowlby and Robertson observed after children returned home from hospitalization.

The breakdown of preoccupied fixation with the caregiver, Bowlby (c. 1965, PP/BOW/D.3/38) noted, usually became dysregulated rage and/or despair. Bowlby’s remarks were primarily based on James Robertson’s observation of hospitalized children on their return home (e.g. 1953; Robertson, 1958). They may also have been influenced by the observations of Bowlby’s friend Robert Hinde, who had found that if infant rhesus monkeys repeatedly threw tantrums that failed to attract the availability of their parent, the infants would intersperse violent jerks of the body with distress calls or orient away from the parent to lie flat and screech (Hinde & Spencer-Booth, 1967). In his later writings commenting on the Ainsworth “resistant” category of Strange Situation behavior, Bowlby (1973, p. 228, 1982, p. 671) observed that anger may be regarded as organized and functional when it is primarily oriented towards achieving the attentional availability of the caregiver; however, he also argues that anger can disorganize a child if its shapeless intensity leads them to lose track of the environment. Today, the meaning of the correlation between the resistance and disorganization scales for the Strange Situation is not yet known. Some researchers think that this correlation is caused by the fact that ultimately they both reflect a single dimension of “anxious” attachment (e.g. Fraley, Roisman, Booth-LaForce, Owen, & Holland, 2013). Others, however, contest this conclusion (e.g. Solomon et al., 2017), though other possible reasons for the association have not yet received adequate discussion in print.

Bowlby’s ideas offer deeper understanding of the manifestations of disorganization and the underlying causes within the attachment behavioral system. Humans begin with the key social elements of attention, expectation, affect, and behavior, which ultimately manifest into a mature attachment system given the availability of adequate caregiving. Additionally, Bowlby’s ideas offer insight into the concept of integration of mental systems, coinciding with interpersonal neurobiology. For Bowlby, the integration of attention, expectation, affect, and behavior is critical to the development and consolidation of behavioral strategies to meet the needs of the attachment behavioral system. These systems can be undermined and, ultimately, be expected to lead to disorganized behavior in the Strange Situation, particularly in infant experiences containing threat conflict, safe haven ambiguity, and/or activation without assuagement.

## Bowlby’s theory: segregated systems

“Segregated systems” is among the most significant concepts of Bowlby’s later work (e.g. 1980; Bowlby, 1988). This concept grew out of his thinking about behavioral disorganization, which he argued was “related to the parallel process in the cognitive sphere … and that a disturbance in the one will create repercussions in the other” (1958a, pp. 365–6), foreshadowing similar assertions by Main and colleagues (1985). It was in thinking about this process that Bowlby developed his concept of segregated systems, which provided a framework for his thinking. It receives a disorientingly short chapter in *Loss* (1980), though the concept organizes much of the book. The idea of segregated systems similarly seems to be pulling the strings in his late essays (e.g. 1988). Following this emphasis, some attachment theorists have used segregated systems as the basis for their thinking and design of attachment measures, such as George and West’s (2012) *Adult Attachment Projective*, which uses segregated systems as the theoretical basis for the adult attachment classification equivalent of disorganization. Despite its clear importance for his thinking, however, Bowlby offered little published discussion of the concept of segregated systems. However, the Bowlby archive contains an unpublished monograph on the subject, entitled *Defences that follow loss: Causation and function* from 1962, written 18 years before the concept appears in print (c. 1962, PP/BOW/D.3/78). The monograph will feature in the forthcoming edited volume of Bowlby’s unpublished writings. The following discussion will link this monograph to Bowlby’s published works to identify how they are connected.

### Disorganization in the context of defense

An animating question of *Defences that follow loss: Causation and function* (Bowlby, c. 1962, PP/BOW/D.3/78) was how to conceptualize disorganization in relation to defense. For Bowlby, a problem arose from the fact that the ethological and psychoanalytic literature differed on where to draw the line between the defense and disorganization. Bowlby was influenced by both schools of thinking and wanted to work at the intersection of these approaches. For instance, ethologists discussed forms of behavioral avoidance, such as looking away, and how animals use such strategies to handle potential threat and/or conflict (e.g. Hinde, 1970). Bowlby thought psychoanalysts would likely agree. However, he felt that the psychoanalytic orthodoxy of his day would conceptualize as *defense* processes that ethologists regarded as indications of *breakdown*, such as alternating between activities or dissociative fugue. Thus, both groups agreed on the description of the behavior, but their interpretations appeared different to Bowlby. He was particularly concerned that an undifferentiated use of the term “defense” among psychoanalysts provided no basis for distinguishing degrees of control: “The relation of defense to healthy control, or to coping processes, has never been clarified. Like Melanie Klein, most analysts hold the view that there are no great differences between them” (Bowlby, c. 1962, PP/BOW/D.3/78). Bowlby acknowledged that some psychoanalysts, like Donald Fairbairn (e.g. 1929), were making distinctions in this area, considering differences between “primitive” and more “mature” defenses. He did not mention Klein’s distinction between the primitive paranoid-schizoid position and the later depressive position, apparently not seeing this distinction as relevant to the kind of thinking he wanted to pursue regarding defense and individual adaptation. To Bowlby, the greater current of psychoanalytic thought, including that of Klein and her followers, directed attention away from the question of which defenses were able to contribute to individual coping, for instance through offering short-term adaptation to an adverse environment for an individual (Bowlby, c. 1962, PP/BOW/D.3/78).

In pursuing this question of how to conceptualize disorganization in relation to defense, Bowlby (c. 1962, PP/BOW/D.3/78) reflected in depth on Freud’s (1915/2001) concept of repression. These unpublished remarks on metapsychology are of particular interest, as they do not have a ready equivalent in Bowlby’s published works. Bowlby fully agreed with Freud that parts of the mind could be separated from one another, but he situated this in the broader context of processes that lead attention to become narrowed away from particular internal or external objects. He asserted “the process of repression is regarded as a special example of the way attention is narrowed during concentration, and the process of overcoming resistance during therapy with that of broadening it again” (Bowlby, c. 1962, PP/BOW/D.3/78).

Bowlby introduced “segregated systems” as an alternative to the traditional term “repression”: “I am introducing the generic term ‘to segregate’ and ‘segregated process’; they denote any process that creates barriers to communication and interaction between one psychic system and another” (Bowlby, c. 1962, PP/BOW/D.3/78). He suggests types of repression, including isolating and undoing, as examples of segregating processes. Bowlby drew on work by Jahoda to present the opposition between integration and segregation as the criterion for distinguishing between healthy and unhealthy forms of coping. Healthy adaptation to adverse environments could be discerned when an organism maintained “integration based on free communication and interaction between different parts of the mental apparatus” (see Jahoda, 1958). For Jahoda, integration of the personality entailed “1) a balance of psychic forces; 2) a unifying (cognitive) outlook; or, 3) a resistance to stress” (Bowlby, c. 1962, PP/BOW/D.3/78). Thus, the most important risk of segregation that Bowlby saw was that forms of attention, expectation, affect, and behavior, or even a whole behavioral system, could fall out of effective communication within the person or with the outside world. As a consequence, opportunities for the internal or external feedback that is so crucial to system functioning would be lost. Bowlby considered that this produces the phenomenon that Freud (1915/2001, p. 187) described as the absence of time or sequencing in the unconscious, which allows children’s segregated wishes or fears to remain potent and mismatched with other experiences, even into adulthood.

### Degrees and forms of segregation

Bowlby’s (c. 1962, PP/BOW/D.3/78) account of segregated systems drew a spectrum between full integration and lack of integration, with different defenses placed along that line. This spectrum of degrees and forms of segregation provided a subtler way of conceptualizing defense mechanisms. Other psychoanalytic thinkers, including Fairbairn (1929), had already distinguished dissociation as a more extreme defense than avoidance. Bowlby’s position took this recognition further in theorizing segregation as a *response* to extremity, a position that would be implicit in his subsequent writings but never elaborated explicitly. On the one hand, mechanisms of defense were conceived by Bowlby (c. 1962, PP/BOW/D.3/78) to arise in situations in which “the integrative function has failed or is about to fail.” In these situations, stress is placed upon mental processes to the point that homeostasis becomes very costly or impossible to maintain, resulting in disorganization for a time. On the other hand, defenses *themselves* enact a weakening of integration by segregating forms of attention, expectation, affect, and behavior. They do so when the alternative might otherwise be greater or more enduring disorganization. As such, defenses have the potential to be *both the cause and result* of integrative failure, via different processes. Defenses, then, permit a certain kind of resilience in the face of disintegrative threats precisely by accepting some determinate and limited degree of segregation. This spectrum of defensive responses demonstrates the degree to which mental integration can vary and the ways in which defensive disruptions to integration can manifest psychologically and behaviorally.

In Bowlby’s (c. 1962, PP/BOW/D.3/78) account, a process such as dissociation would not be regarded as mere breakdown (following the ethologists) nor as a well-orchestrated defense (following Bowlby’s view of psychoanalytic orthodoxy at the time). Instead, dissociation is conceptualized as a far point on the spectrum of segregation of mental processes – an emergency response to the near threat of disorganization. In the years after Bowlby was writing, it is notable that clinical dissociation was found to be one outcome associated with disorganized attachment (see Sroufe et al., 2005), though some forms of disorganization may certainly be more linked to dissociative processes than others (Carlson, Yates, & Sroufe, 2008; Hesse & Main, 2006). The link between disorganized attachment and clinical dissociation is an important example of the relational development of nonintegrated states becoming nonintegrated traits of the individual (Graziano, 2014; Siegel, 2012). The disorganization of attachment processes can impact the very experience of focal attention, which is how the mind organizes consciousness through processing of experience, energy, and information; it therefore has some similarities in mechanism to psychological trauma, without the two being reducible to one another (Fearon, 2004; Siegel, 2017).

This process of mental segregation in the context of threats to integration might be a source of the chaotic and catastrophic fantasies and representations of self and other discerned by researchers studying the sequelae of infant disorganized attachment in middle childhood (e.g. Main et al., 1985; cf. Solomon & George, 2016; Solomon, George, & De Jong, 1995). Attachment in middle childhood is often assessed using doll play, which presents scenarios of danger and asks the child to finish the story. One of the patterns produced by children who are disorganized is chaotic and catastrophic fantasies. This is understood to indicate that the disorganization that is observable in infant behavior has begun to shift to the representational level in middle childhood, which may occur, at least in part, due to the segregation of mental processes proposed by Bowlby (c. 1962, PP/BOW/D.3/78).

Bowlby (1973, 1980, c. 1962, PP/BOW/D.3/78) thought of non-dissociative defenses as less emergency measures. Again, this is a position that is implicit but not elaborated explicitly in his subsequent writing. Avoidance, for instance, has a variety of forms and degrees. It can range from the simple reallocation of attention away from distress to more substantial forms that result in limited segregation by diverting attention to something else. For instance, intrusive parenting is associated with avoidance in the Strange Situation, likely because the infant attempts to shuts down their attentional availability to their parent where otherwise the parent’s interactions with them would be overwhelming (Isabella & Belsky, 1991; Sroufe, 1996). It is notable that an avoidant attachment classification in the Strange Situation made a smaller but independent contribution over and above disorganization to dissociative behaviors in late adolescence in the Minnesota Longitudinal Study (Sroufe et al., 2005). Avoidance is a rigid, brittle form of organization with significant disadvantages, such as not seeking help when needed or even registering the need for help. However, for Bowlby in his unpublished writings, as later for Main (1979), avoidance does not in itself undermine organization at the level of the attachment system. Defenses that are less radical and more flexible present lower levels of long-term threat to mental health and may even be beneficial in the short term (see also Bowlby, 1980, p. 64), though of course much depends on for how long and how intensely they are sustained and in what context.

## Bowlby’s theory: defensive exclusion

A key aspect of Bowlby’s thinking about disorganization, defense, and segregation was that different kinds of defenses and their varying degrees could be distinguished by the extent of segregation that resulted. Interpersonal Neurobiology today would define this as the *degree of impediment to integration* (see Siegel, 2017). Bowlby (c. 1962, PP/BOW/D.3/78; cf. seminar by Bowlby delivered at the Tavistock on February; 1958, PP/BOW/H.67) emphasized that holding incompatible models and expectations within parts of the mind that are firmly segregated, and thus unable to communicate with each other, can threaten successful functioning. Some incompatibility in the psyche is an inevitable part of being human and localized and controlled incompatibility can provide a foundation of fantasy, creativity, and work–life balance, which can feel quite freeing. However, without communication and feedback between systems, and thus perceptions of the world, effector equipment cannot orchestrate the systems in a coherent manner that is responsive to the environment. Like the sole of a shoe, some limited and strategic segregation can save us from the over-exposure of walking barefoot through the world, but when the sole is too thick, we lose the chance for the information and balance gained from our sensed contact with the ground.

### Fantasy

Bowlby (c. 1962, PP/BOW/D.3/78) accepted the basic psychoanalytic axiom that some segregation was inevitable within and between behavioral systems, and hence within and between the representations of self and other held by those systems. For instance, he was mindful that both defenses and disorganization might be shaped not only by present circumstance but also by expectations, fears, and wishes evoked by, but not reducible to, past experience. These come trailing any present behavior like the tail of a comet and, in Bowlby’s account, comprise the domain that psychoanalysts term *fantasy*.

Fantasy is largely missing from Bowlby’s published works but is given considerable attention in his unpublished book, *Defences that follow loss: Causation and function* (Bowlby, c. 1962, PP/BOW/D.3/78). There he states:

It will be noted that in referring to different sorts of behaviour I have each time added in brackets ‘with its associated affects and fantasies’. The reason is that I conceive overt behaviour to be only one component of a motivational system within the organism, and fantasies, thoughts and affects, conscious and unconscious, to be integral to, and other components of, such systems.

Bowlby acknowledged that there is something potentially creative and freeing in the gap of potential incompatibility between felt and historical experience that fantasy represents. Frightening intensities of incompatibility, however, can result in mental segregation if the experience of fright is strong enough, producing the symptomatic responses that Bowlby saw in his patients following trauma. For instance, in his work with war veterans from World War II, he saw how the symptoms he was observing had roots in the deep, regular, and knotted conflict these individuals had sustained between a desire to flee in fear and a sense of duty and camaraderie (War Neurosis Memorandum, 1940, PP/BOW/C.5/1). The intensity and the rigidity of the conflict between these two responses, and the extremity and rigidity of the defenses used to manage the conflict, had led to the symptoms shown by these patients. Thus, flexibility in the capacity to draw upon and utilize defenses can be key to understanding how incompatibility affects attention, expectation, affect, and behavior.

### Selective exclusion

Among the defenses he had observed clinically, Bowlby (c. 1962, PP/BOW/D.3/78) was particularly interested in the way that historical events could be kept from conscious attention. He used the term *selective exclusion* to refer to the way in which attention divides the field of awareness into relevant and irrelevant, imaginable, and feasible. This process segregates consciousness from many of those aspects regarded as irrelevant, allowing us to mentally exclude certain associations and information. There is always some level of exclusion in human experience. However, Bowlby thought that long-term mental health would be supported by effective communication between mental systems on the basis of relative and flexible forms of segregation, rather than those that were strictly held. This position has found considerable support in the decades since Bowlby was writing (e.g. Siegel, 2017). The direction and integration of attention, expectations, affect, and behavior need not be the same across all the domains of life by any means, from play to work to idling to affectionate relationships. Variation is expected and can be beneficial. For Bowlby, the potential for communication between different domains of life and mutual enrichment support mental health (Bowlby, c. 1962, PP/BOW/D.3/78). For instance, selective exclusion could be helpfully used to keep worries away during relaxation or sleep. The direction and quality of attention would need to be flexible enough to change once work began again. However, where this can be achieved, communication between systems ensures that benefits of physical and attentional rest were transferred in the form of feeling genuinely refreshed. Bowlby’s account provides a place for localized and flexible segregation and even highlights its potential benefit.

One potential benefit of selective exclusion is to avoid overload and unhelpful discrepancies so as to maintain integration. Bowlby (c. 1962, PP/BOW/D.3/78) elaborated the role of selective exclusion in the context of information integration, arguing that

information of any sort that is incompatible with existing information, or motivation that is inconsistent with existing motivation, is never welcome. To use and integrate it may require drastic reorganisation of existing schemas and systems; and inevitably this must be preceded by initial disorganisation…. The alternative and more frequent method of responding to incompatible information and motivation is to exclude it. By doing so, disorganisation is made unnecessary and mental pain avoided. (PP/BOW/D.3/78)

This theoretical conceptualization offered Bowlby a means of respecifying the psychoanalytic distinction between conscious and unconscious. Bowlby observed, “consciousness seems to be heightened when selective exclusion is reduced so that more information and a greater variety of actions are together permitted integration” (Bowlby, c. 1962, PP/BOW/D.3/78). Referring to other writers’ works, he states, “Cobb (1952) has suggested that 'it is integration itself, the relationship of one part to another, that is mind and which causes the phenomenon of consciousness; and Fessard (1954) has accordingly proposed that consciousness be termed an ‘Experienced Integration’” (Bowlby, c. 1962, PP/BOW/D.3/78). This perspective on the mind is one that feels resoundingly contemporary and is well aligned with Tononi’s (2012) integrated information theory of consciousness.

Bowlby (c. 1962, PP/BOW/D.3/78) applied his account to the nature of defense, arguing that the process of selective exclusion can also be “exploited” by the organism, forming various kinds of defense. He cites the psychoanalytic theorist and clinician Thomas Morton French (1958) who had proposed that “the normal function of the Ego is its integrative function; defenses are activated only when the integrative function has failed or is about to fail” (p. 32). When integration is threatened, the capacity for selective exclusion can be exploited to produce what Bowlby (c. 1962, PP/BOW/D.3/78) termed *defensive exclusion*, and which he saw as the basic psychological process behind avoidance. This position would be stated years later in *Loss* (1980), but with little account of the underpinning metapsychology. The mental apparatus retains some conditional integration in deploying defensive exclusion in response to an experience that would otherwise be overwhelming, though at the price of segregating certain kinds of environmental information, paralleled by the segregation of mental systems and their neurological architecture. Bowlby argued

there can be no doubt, therefore, that selective exclusion is an integral and ubiquitous part of the action of the CNS [central nervous system]. That the segregating processes characteristic of pathological defence may be special cases of it was, as we have seen, adumbrated by Freud in 1926, though he never elaborated the idea. (Bowlby, c. 1962, PP/BOW/D.3/78)

This conceptualization offers an understanding of how exclusion can shift from being selective to defensive. Emphasizing the importance of these responses for the development of mental illness, Bowlby wrote, “What characterises a pathological condition is that exclusion acts in such a way that it creates not only the usual temporary barrier but a permanent one. Thereby psychic systems are segregated from one another as though by an iron curtain” (Bowlby, c. 1962, PP/BOW/D.3/78). In this way, defensive exclusion can ultimately undermine integration and shift the mind into a segregated state.

### Avoidance as a defense against disorganization

Drawing from his theory of defensive exclusion, Bowlby (c. 1962, PP/BOW/D.3/78) was especially interested in avoidance both as a defense against disorganization and for how it yields to disorganization when overwhelmed. He proposed that prolonged and intense utilization of avoidance could result in the selective exclusion of internal or external cues to relational needs. This could then render the attachment behavioral system difficult to access, and leave individuals unable to know how to even want love and affection, let alone be able to take action to meet their relational needs. Such defensive exclusion can then inhibit the ability to update representational models of self and other, since discrepant experience and information remain segregated and unavailable.

Bowlby (c. 1962, PP/BOW/D.3/78) saw segregation largely as a matter of degree, with some communication maintained between systems even though it might be distorted or incomplete. Even when the segregation is extensive, a subordinated system may still intrude in ways that are neither suited to the behavioral approach of the dominant system nor the demands of the current situation. For example, where there has been segregation of mental systems, a wave of grief, tender affection, or emotional exhaustion might ambush us without obvious cause or elicitation from the present (see Bowlby, 1989). Other examples would be outbursts of angry, distressed, sexual, or caregiving behavior that are direct or indirect expressions of an otherwise segregated system, such as a craving for food that enacts subordinated lines of longing to be cared about. Bowlby (c. 1962, PP/BOW/D.3/78) notes that such outbursts are, generally, “ill organised” and not well-suited to environmental demands, even when they take on an expectable rhythm:

That the motor responses adopted in such conditions of stress tend to become fixated and so lead to pathological behaviour is now fairly well known. What is perhaps less clearly recognised is that the underlying mechanism of selective exclusion itself becomes deranged. Instead of being sensitive, efficient and reversible, it becomes stuck in a condition that is at once restrictive, erratic and rigid. Not only are information and motor response relevant to any one goal narrowly restricted but information and motor responses relevant to some other and perhaps incompatible goal may be allowed through. It is as though an enquiry clerk, when asked about trains to Cornwall, gave information endlessly about the night express to Plymouth, with occasional intrusions about a plane to Rome. (Bowlby, c. 1962, PP/BOW/D.3/78)

The idea of intrusion of excluded and segregated material in inappropriate contexts reappeared much later in Bowlby’s published writings (e.g. 1979, 1980, 1988). He argued

When yearning for love and care is shut away, it will continue to be inaccessible. When there is anger, it will continue to be directed at inappropriate targets. Similarly anxiety will continue to be aroused by inappropriate situations and hostile behaviour be expected from inappropriate sources. (1979/1988, p. 132)

Bowlby expected such responses, especially at “times when fragments of the information defensively excluded seep through so that fragments of the behaviour defensively deactivated become visible” (1980, p. 65).

Defense in the context of segregated systems represents an important theoretical contribution of Bowlby’s that was never expressed fully in publication. Understanding when and how a defense crosses the threshold from adaptive to pathological, such as when selective exclusion shifts to become defensive exclusion, is key to understanding mental segregation. This brings us back to the larger question of thresholds for pathology and offers guidance in how to understand, interpret, and apply this psychological process in empirical and clinical work.

## Implications

This goal of the paper was to illuminate some of Bowlby’s unpublished theories and ideas about what would ultimately be called disorganized attachment by Main and Solomon (1986, 1990). We have also flagged correspondences between Bowlby’s theory of disorganization and current neurobiological ideas regarding the interplay between parent–child interactions and the self-organization of physiological systems. Bowlby saw affective experiences as the source of the attachment behavioral system’s organization and regulation. He used the concept of “effector equipment” to describe how the elements of attention, expectation, affect, and behavior become organized to orchestrate flexible and appropriate responses to the environment. Caregiver availability facilitates this integration. Bowlby theorized about three potential pathways to disorganization: (1) threat conflict, (2) safe haven ambiguity, and (3) activation without assuagement, as they can result in failure to coordinate and integrate across the attention, expectation, affect, and behavior of the attachment system. Indeed, these pathways have found empirical support by later researchers (e.g. Bernard et al., 2012; Bernier & Meins, 2008; Lyons-Ruth, 2007; Main & Solomon, 1990). Further, Bowlby’s unpublished writings add color and detail to his published work on segregated systems and defensive exclusion.

Bowlby’s theory of disorganization has a number of implications for contemporary research and clinical practice. We will highlight a few of these in closing, with the clear caveat that these are speculations and require further empirical exploration.

### Defense, disorganization, and intervention

One notable aspect of Bowlby’s position is that defense is more rigid than disorganization, even though defenses can be useful when dealing with perceived adversity (Bowlby, c. 1962, PP/BOW/D.3/78). Based on his experiences as a clinician working with individuals in the context of mourning and loss, Bowlby (e.g. 1961, p. 325, p. 332, 1980, p. 246; Bowlby, c. 1962, PP/BOW/D.3/78) believed that defense mechanisms like denial can be helpful at times for individuals, and certainly can keep an individual in a comparatively better state than disorganization, at least in the short term. As they develop, children in adverse circumstances generally elaborate strategies and defenses adapted to their caregiving environment. However, Bowlby also argued that clinical interventions might be more effective with individuals experiencing disorganization than those utilizing well-established defenses: essentially, non-organized and nonintegrated states may be less entrenched and more accessible to change than stable and settled defenses.

When thinking about disorganization as a Strange Situation classification, Bowlby’s conclusion may initially seem counterintuitive. Infants with a disorganized relationship are often assumed to be in a less favorable and more stuck position than those classified as organized-insecure: “The insecure disorganized attachment classification which is often associated with early maltreatment is [the] most resistant to change” (Furnivall, McKenna, McFarlane, & Grant, 2012, p. 13). However, there are emerging findings supporting Bowlby’s proposal that interventions will be especially effective for infant–caregiver dyads who have received a disorganized classification. One source of support comes from findings that infant–caregiver relationships classified as disorganized are likely to become secure if they are able to organize in the context of a caregiving intervention for the parent(s) (Bernard et al., 2012). Additionally, though not based on an intervention, Wang, Willoughby, Mills-Koonce, and Cox (2016) observed that children who received a disorganized attachment classification in infancy but experienced high levels of maternal sensitivity in toddlerhood showed greater decreases in externalizing behavior across this period than those classified as insecure but organized in infancy. It will be important for future research to continue to empirically examine the stability of the disorganized attachment classification in the context of intervention, and its comparative responsiveness to intervention efforts.

### Different forms of disorganization

A second implication of this paper is the relevance of Bowlby’s thinking about different forms of disorganization in infancy. Hesse and Main (2006) have argued that it would be “a worthwhile endeavor for developmental psychopathology” to study different caregiving contexts and “compare these to the forms of D behavior exhibited by their infants” (p. 335). They indicate that some forms of disorganized behavior described in the Main and Solomon (1990) indices seem to have a dissociative mechanism, some suggest manifest fear of the caregiver as their mechanism, while still others indicate more diffuse states of conflict about approaching the caregiver. Similar calls to consider differences among children classified as disorganized have been heard from other researchers in recent years (e.g. Lyons-Ruth & Jacobvitz, 2016; Solomon et al., 2017).

Bowlby’s unpublished reflections have value for the development of hypotheses for such inquiry. In the unpublished discussions described here, Bowlby differentiates between the disorganization that may occur in the context of avoidance versus in the context of resistance. Bowlby directs attention towards potential differential associations between the indexed behaviors and the Ainsworth patterns, based on differences in the child’s experience. For instance, his thinking suggests that abrupt intrusions made by segregated affects or tension behaviors would be more associated with avoidance than other patterns of attachment. This is an implication of Bowlby’s position that has also been drawn by Main and Hesse (1992) based on Bowlby’s published work. The unpublished manuscripts available in the Bowlby Archive suggest that this predicament will occur when a child’s experience has led them to adopt avoidance as a conditional strategy but the degree of conflict between distress and avoidance undermines the effector equipment that would usually coordinate behavior and affect in a coordinated manner. Comparison of the Main and Solomon indices with the Ainsworth resistance and avoidance scales could be readily conducted on already existing datasets. This would be of particular clinical interest in terms of understanding different processes involved in disruption of the attachment system, as well as wider aspects of emotional dysregulation in young children.

### Effector equipment and self-regulation

A final point we wish to draw out from Bowlby’s theorizing is the significance of effector equipment (1969; Bowlby, c. 1962, PP/BOW/D.3/78), which might now be termed executive function or self-regulation. Disorganization in middle childhood is often assessed using representational measures such as picture or story-stem tasks that provide narratives about family interactions, and the production of these narratives in part taps the child’s capacity for self-regulation (Solomon & George, 2008). It is notable that pharmacological treatment of children for attention-deficit hyperactivity disorder (ADHD) has been found to apparently eliminate the disorganized classification as measured by children’s representations (Storebø et al., 2014). Though it is important to note that they had a small sample, Storebø and colleagues (2014) found that all of the children diagnosed with ADHD who were initially classified as disorganized and received medication as their only treatment were no longer classified as disorganized 6 months later (Storebø et al., 2014). This raises the question of whether the attachment system had *truly* organized or whether the expression of attachment through representation had somehow been *masked*.

One clue from cross-sectional research indicates that the link between disorganized attachment and difficulty with attention may be rooted in dysregulated emotionality (Forslund, Brocki, Bohlin, Granqvist, & Eninger, 2016). Given Bowlby’s theory, it might be that pharmacological support for the functioning of effector equipment increases the coherence of attention, expectation, affect, and behavior, thus reducing the expression of disorganization, at least for forms that can be assessable using representational measures. However, this process should be distinguished from actually reducing the overall disorganization of the attachment system, which is a product of segregated systems. These concerns tap into larger questions about the connection and potentially parallel development of self-regulation and attachment. Ongoing and future longitudinal research on infant disorganized attachment behaviors and later ADHD symptomology will help answer these questions.

## Conclusion

This paper examined Bowlby’s unpublished writings and reflections on the development and organization of attachment. Bowlby (1988) emphasized the importance of distinguishing between the *context of discovery* and the *context of justification*, following Karl Popper. The context of discovery refers to the conjecture and presentation of ideas, whereas the context of justification is the attempt to falsify an idea by amassing evidence – strong support comes from the repeated failure of the data to falsify the idea. This paper, relating speculations in Bowlby’s manuscripts and notes, is firmly grounded in the context of discovery. Much of this information has not been previously published, let alone tested, and interpretations and applications of these ideas should be considered in that light. It is our hope that the remarks presented here will support future research and clinical thinking about the nature of attachment, self-regulation, and defense.

## Appendix

Timeline of Bowlby’s reflections on disorganized attachment processes and behaviors

**1939** **–** **1942**

- Bowlby works on unpublished manuscripts describing the behavior of evacuated children (PP/BOW/C.5/4/1).

**1940**

- Bowlby and Soddy write “War Neurosis Memorandum” including descriptions of the conflicted and dissociative behaviors of combat veterans fromthe Second World War (PP/BOW/C.5/1).
- Bowlby publishes “Influence of early environment in the development of neurosis and neurotic character” in the *International Journal of Psycho-Analysis.*

**1944**

- Bowlby publishes “Forty-four juvenile thieves” in the *International Journal of Psycho-Analysis.*

**1951**

- Bowlby publishes *Maternal Care and Mental Health* for the World Health Organization (WHO).
- Robertson and Bowlby begin writing notes describing what they term “panic responses” in children on return from hospitalization (PP/BOW/D.3/1).

**1952**

- Bowlby, Robertson, and Rosenbluth publish “A two-year-old goes to hospital” in *Psychoanalytic Study of the Child.*

**1953–1957**

- Bowlby accumulates extensive unpublished file-draw notes integrating psychoanalytic theories of conflict with ethological observations of conflict in animals. This results in the 1957 publication of “An ethological approach to research on child development” in the *British Journal of Medical Psychology*.

**1960**

- Bowlby publishes articles on “Separation anxiety” and “Grief and mourning in infancy and early childhood” in the *International Journal of Psycho-Analysis.*

**1962**

- Bowlby works on “Defences that Follow Loss: Causation and Function,” which remains unpublished (PP/BOW/D.3/78).

**1965**

- Bowlby and Robertson complete a version of *Protest, Despair and Detachment*, which remains unpublished (PP/BOW/D.3/38).

**1966**

- Hinde publishes *Animal Behavior*, offering a theory of conflict behavior that will be influential for both Bowlby and Main (see Solomon et al., 2017).

**1969**

- Bowlby publishes *Attachment*, volume 1.

**1973**

- Bowlby publishes *Separation*, volume 2 of his trilogy. The chapter “Forms of behaviour indicative of fear” discusses conflict and disorganization and critically examines ambiguities in usage of the term “fear,” drawing particularly from two unpublished manuscripts: “Types of fear response” (1968) and “Wariness” (1973).
- Mary Main graduates with a PhD in Psychology from The Johns Hopkins University. During her dissertation, she asked her undergraduate coders to make particular note of any “odd” behavior shown by infants.

**1978**

- Ainsworth and colleagues publish *Patterns of Attachment.*
- Bowlby watches Strange Situation tapes with Mary Main and they discuss observations of conflict behavior (PP/BOW/H.78).

**1979**

- George and Main publish “Social interactions of young abused children” in *Child Development.*
- First use of a D category by Judith Solomon in coding notes for the Strange Situation in Main’s Berkeley laboratory.

**1980**

- Bowlby publishes *Loss*, volume 3 of his trilogy.

**1981**

- Main and Stadtman publish a study of conflict behavior “Infant response to rejection of physical contact by the mother: Aggression, avoidance and conflict” in the *Journal of the American Academy of Child Psychiatry.*

**1986**

- Main and Solomon publish their chapter on the “Discovery of an insecure-disorganized/disoriented attachment pattern.”

**1988**

- Bowlby approves Main and Solomon’s new disorganized category in *A Secure Base*.

**1990**

- Main and Solomon publish the coding protocols for disorganized attachment.
- John Bowlby passes away at the age of 83.
